# Two CONSTANS-LIKE genes jointly control flowering time in beet

**DOI:** 10.1038/s41598-018-34328-4

**Published:** 2018-10-31

**Authors:** Nadine Dally, Maike Eckel, Alfred Batschauer, Nadine Höft, Christian Jung

**Affiliations:** 1 UKSH Campus Kiel, Hematology Laboratory Kiel, Langer Segen 8-10, D-24105 Kiel, Germany; 2 Plant Breeding Institute, Christian-Albrechts-University of Kiel, Am Botanischen Garten 1-9, D-24118 Kiel, Germany; 3 Department of Plant Physiology and Photobiology, Faculty of Biology, Philipps-University of Marburg, Karl-von-Frisch-Str. 8, D-35032 Marburg, Germany

## Abstract

Breeding vegetative crops (e.g. beets, cabbage, forage grasses) is challenged by two conflicting aims. For field production, flowering must be avoided while flowering and seed set is necessary for breeding and seed production. The biennial species sugar beet makes shoot elongation (‘bolting’) followed by flowering after a long period of cold temperatures. Field production in northern geographical regions starts in spring. A thickened storage root is formed only during vegetative growth. It is expected that winter beets, which are sown before winter would have a much higher yield potential. However, field production was not possible so far due to bolting after winter. We propose a strategy to breed winter beets exploiting haplotype variation at two major bolting time loci, *B* and *B2*. Both genes encode transcription factors controlling the expression of two orthologs of the Arabidopsis gene *FLOWERING LOCUS T* (*FT*). We detected an epistatic interaction between both genes because F_2_ plants homozygous for two *B/B2* mutant alleles did not bolt even after vernalization. Fluorescence complementation studies revealed that both proteins form a heterodimer *in vivo*. In non-bolting plants, the bolting activator *BvFT2* was completely downregulated whereas the repressor *BvFT1* was upregulated which suggests that both genes acquire a *CONSTANS* (*CO*) like function in beet. Like CO, B and B2 proteins house CCT and BBX domains which, in contrast to CO are split between the two beet genes. We propose an alternative regulation of *FT* orthologs in beet that can be exploited to breed winter beets.

## Introduction

The transition from the vegetative to the generative phase is of major interest to crop breeders due to its high relevance for yield and quality. Crop plants show great variation regarding their phenological development. If vegetative parts of the plant are harvested (leaves, roots) they must not enter the reproductive phase, a major step in plant development commonly referred to as floral transition. Sugar beet (*Beta vulgaris* L.) is a typical vegetative crop with a biennial life cycle. After sowing in spring, it produces huge leaf and root mass until harvest in autumn. As a result of secondary thickening, a storage root is produced with sucrose contents between 17–20%^1^. As a biennial plant it enters the reproductive phase only after exposure to a long period of cold temperatures (<4 °C). Then, the shoot is elongated (‘bolting’) and flowers are produced. Early bolting under field conditions must be strictly avoided because it gives rise to flowering plants with small roots and low sucrose content. For seed production, plants must bolt and flower early after winter. This follows, that conventional sugar beet cannot be cultivated as a vegetative crop over winter, commonly referred to as ‘winter beet’^1^.

Quantitative trait loci (QTL) and major genes controlling bolting time have been mapped to the nine beet chromosomes^2^. The bolting time QTL *SEASONAL BOLTING- 4* and -9 (*SBT-4, SBT-9*) accounts for up to 52% of the phenotypic variation^3^. The phenotypic effect of *SBT-4* is likely caused by the major flowering time regulator *BvFT2* because they were mapped to the same position on chromosome 4. *SBT-9* was precisely mapped to the position of *BR1*. This QTL was recently fine mapped by a sequencing approach and a gene similar to *CLEAVAGE AND POLYADENYLATION SPECIFICITY FACTOR 73-I* (*CPSF73-I*) from Arabidopsis was suggested as a candidate gene for this QTL^4^.

Sugar beet has two sequences which share high homology to *FLOWERING LOCUS T* (*FT*) a major integrator of signals from different regulatory pathways triggering floral transition in Arabidopsis^5^. *BvFT1* is a floral repressor which is transcriptionally active before winter and prevents bolting. In contrast, *BvFT2* is a floral inducer which is activated during vernalization. A high *BvFT2* activity is indicative for generative (bolting) beet plants^5^.

Two upstream regulators of the two *BvFT* orthologs have been cloned. *BOLTING TIME CONTROL 1* (*BTC1*) belongs to the *PRR3/7* clade of *PSEUDO RESPONSE REGULATOR* (*PRR*) genes that are components of the photoperiod pathway in Arabidopsis^6^. A dominant allele which is highly abundant in wild beet (*B. vulgaris* ssp. *maritima*) populations from the Mediterranean causes early bolting (without vernalization) resulting in an annual life cycle. Another *PRR7* homolog, *BvPRR7*, is a cold responsive gene with a clock function in beets but not involved in bolting time regulation^7^. The second bolting time gene, *BvBBX19* encodes a putative transcription factor with two B-Box zinc finger motifs but lacking a CCT domain^8^. Recently, haplotype variation of the four major bolting time genes from beet have been studied in wild and cultivated beet accessions^9^. For *BTC1* and *BvBBX19*, 14 and 7 haplotypes were found, respectively^6,9,10^. They were classified as annual or biennial bolting time regulators. *BTC1* and *BvBBX19* share homology with the transcription factor *CONSTANS* (*CO*), which regulates floral transition in Arabidopsis in a long day (LD) dependent manner^11^. It has two consecutive Zn finger domains which are called B-Boxes^12^. Mutants with amino acid alterations in conserved residues of the B-Boxes are late flowering. At the C-terminus, the CO protein has a CCT (CO, CONSTANS-LIKE, and TIMING OF CAB EXPRESSION1) domain which includes a nuclear import signal. By its CCT domain, CO binds to the ubiquitin ligase COP1 and to the *FT* promoter by forming complexes with other transcription factors^13^. This sequence is strictly conserved in proteins which are constituents of the circadian clock^12^. CDF (CYCLING DOF FACTORS) transcription factors bind to the *CO* promoter and inhibit its expression during the morning. Later, they are degraded by the proteasome when GIGANTEA (GI) interacts with FLAVIN BINDING, KELCH REPEAT, F-BOX PROTEIN 1 (FKF1) and ZEITLUPE (ZTL) resulting in strong transcriptional upregulation of *CO*^14^. The CO protein is stabilized by light and degraded in darkness after ubiquitination and proteolysis by the 26S proteasome^15^.

In Arabidopsis, apart from *CO* there are at least 31 genes encoding proteins with B-Box and CCT domains, 16 are *CO*-Like (COL) proteins with one or two B-Boxes and one CCT domain, the remaining ones are either lacking the CCT domain, or one B-Box and the CCT domain^16^. BBX19 and CO are forming dimers which jointly regulate *FT* in an antagonistic way^17^. BBX32 physically interacts with COL3 to form a dimer which targets the *FT* promoter^15^. Interestingly, beet has a large CONSTANS-LIKE gene family but is lacking a functional *CO* ortholog with both domains^18^. *BTC1* is lacking a B-Box and *BvBBX19* is lacking a CCT domain.

The purpose of this work was to understand the genetic and physical interaction between *BTC1* and *BvBBX19* and to lay the foundations to breed winter beets. We assumed that both proteins work together to acquire a CO-like function. To test our hypothesis, we studied an F_2_ population segregating for both genes. We found an epistatic interaction between both loci which resulted in three different life cycle regimes. Combining two mutant alleles resulted in plants which completely lost their competence to bolt after vernalization. The genetic data were confirmed by yeast-two-hybrid interaction and *in planta* bimolecular fluorescence complementation studies. Double mutant plants are proposed as prototypes for winter beet breeding which requires complete bolting control after winter.

## Results

### The *B2* locus is epistatic to *B*

We produced an F_2_ population from a cross between two biennial beet genotypes, seed code 093187 (*B*_*a*_*B*_*a*_ *B2*_*f*_*B2*_*f*_) and 056822 (*B*_*d*_*B*_*d*_ *B2*_*h*_*B2*_*h*_) which differed by their *B* and *B2* alleles. 145 plants were grown under long day conditions together with their parents and the annual and biennial controls. We determined the genotypes of the *B* and *B2* loci for all F_2_ plants using the markers CAU4234 and CAU4235 (Supplementary Tables 1 and 2). In accordance with their position on different chromosomes, the observed genotypic segregation fitted a random segregation ratio (χ^2^ = 15.77; α = 0.05) (Supplementary Tables 3 and 4).

**Figure 1:**
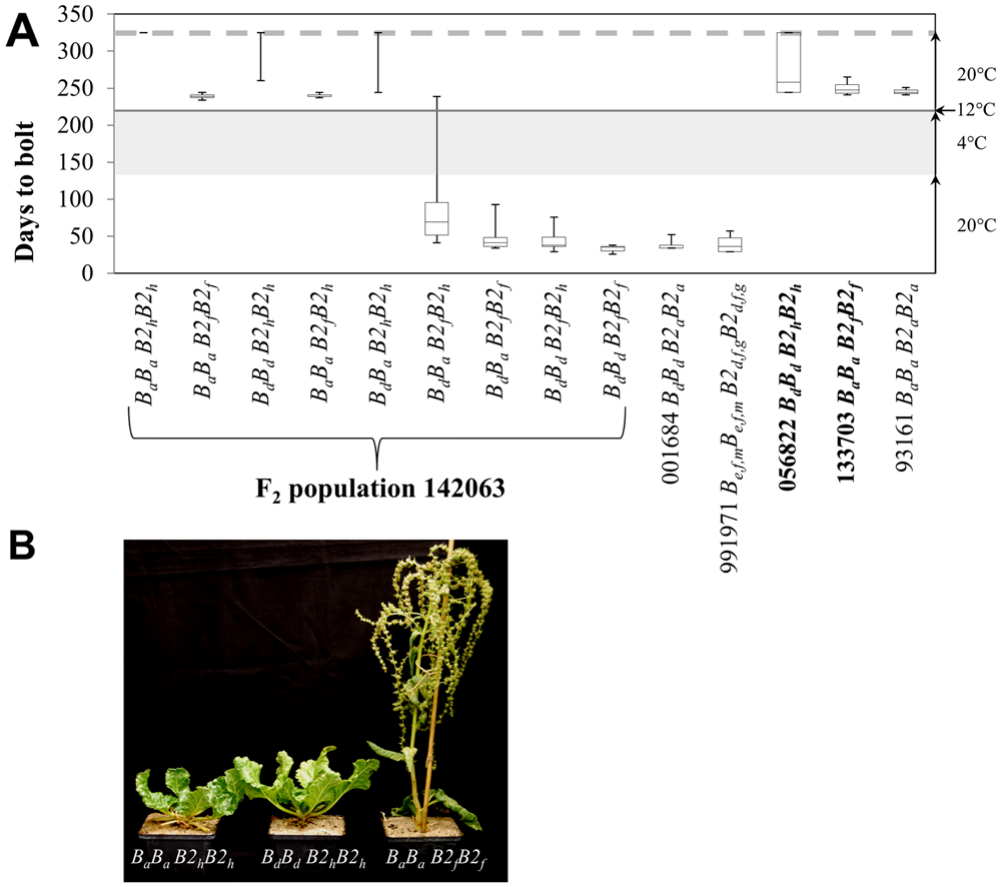
Phenotypic variation for bolting time in the F_2_ population. Box plots indicate the first and third quartile range of the phenotypic variation for each genotype. The top and bottom whiskers indicate the maximum data value. (**A**) F_2_ plants (seed code 142063) and annual (001684, 991971) and biennial (056822, 133703, 930176) controls were grown in a climate chamber under long day conditions (16 h light/8 h dark) at 20 °C. After 135 days, non-bolting plants were kept for 12 weeks at 4 °C. For plants which did not start to bolt until the end of the experiment (grey dotted line), days to bolting was set at 325. The respective genotypes for *BTC1* (*B*) and *BvBBX19* (*B2*) are given according to Höft *et al*.^9^. Seed code 056822 and 133703 are the parental genotypes (bold letters). (**B**) Three F_2_ plants with their respective genotypes after 12 weeks of cold treatment.

The biennial controls bolted within 3–4 weeks after vernalization whereas the annual controls bolted early (4–6 weeks after sowing) without vernalization required (Fig. 1A). Most F_2_ plants carrying at least one *B*_*d*_ and one *B2*_*f*_ allele were lacking a vernalization requirement because they bolted within 114 days after sowing like plants from the annual controls (Fig. 1A). Six F_2_ plants with the *B*_*d*_*B*_*a*_ *B2*_*f*_*B2*_*h*_ genotype did not bolt prior to cold treatment. However, these plants were the earliest biennials as they bolted already 9–16 days after vernalization. Neither the F_2_ genotypes carrying the homozygous *B*_*a*_ allele in combination with *B2*_*h*_ or *B2*_*f*_ alleles (*B*_*a*_*B*_*a*_ *B*_*f*_*B2*_*f*_, *B*_*a*_*B*_*a*_ *B2*_*f*_*B2*_*h*_, *B*_*a*_*B*_*a*_ *B2*_*h*_*B2*_*h*_) nor those plants carrying the *B*_*d*_ allele (heterozygous or homozygous) in combination with the homozygous *B2*_*h*_ allele (*B*_*d*_*B*_*d*_ *B2*_*h*_*B2*_*h*_, *B*_*d*_*B*_*a*_ *B2*_*h*_*B2*_*h*_) were able to bolt without cold treatment. After vernalization, all F_2_ plants homozygous or heterozygous for the *B2*_*f*_ allele (*B*_*a*_*B*_*a*_ *B2*_*f*_ *B*_*f*_, *B*_*a*_*B*_*a*_ *B2*_*f*_*B2*_*h*_) started shoot elongation within three weeks which is typical for biennial beets.

Consistent with our initial hypothesis, the F_2_ population displayed a third phenotypic class for bolting time because 27 out of 30 F_2_ plants that carry the homozygous *B2*_*h*_ allele in combination with the homozygous and heterozygous *B*_*d*_ allele and all 17 *B*_*a*_*B*_*a*_ *B2*_*h*_*B2*_*h*_ F_2_ plants failed to bolt until the end of the experiment (325 days after sowing). The fact that almost all F_2_ plants carrying the homozygous *B2*_*h*_ allele were non-bolting after vernalization irrespective of the *B* allele indicates that the *B2*_*h*_ allele is able to ‘mask’ the phenotypic effect of the *B*_*d*_ or *B*_*a*_ alleles.

How can transgressive variation in the F_2_ population be explained? We tested two genetic hypotheses to explain the phenotypic segregation observed in this experiment (Supplementary Table 4). Our initial hypothesis follows the assumption that all plants carrying at least one *B*_*d*_ and *B2*_*f*_ allele are annual, plants which are homozygous for either the *B*_*a*_ or *B2*_*h*_ allele are biennial, and only the double homozygous F_2_ plants (*B*_*a*_*B*_*a*_ *B2*_*h*_*B2*_*h*_) do not bolt after vernalization giving rise to a phenotypic segregation of 9:6:1 (annual: biennial: non-bolting after vernalization). This hypothesis was rejected after a χ² test for goodness of fit to a 9:6:1 ratio (χ^2^ = 150.03; α = 0.01). The second hypothesis is based on the assumption that the *B2*_*h*_ allele acts epistatically over the *B* locus. In this case, a 9:4:3 phenotypic segregation was to be expected (Supplementary Table 4). As this segregation rate was not rejected (χ^2^ = 2.24; α = 0.01), we assume that the *B2*_*h*_ allele which was derived from an EMS mutagenesis acts epistatically to *B* resulting in a non-bolting (after vernalization) phenotype (Supplementary Table 4). However, this interaction does not fully explain phenotypic variation because biennial plants were found in the *B*_*d*_*B2*_*h*_ parent 056822 and among the corresponding F_2_ genotypes (Fig. 1A). In conclusion, genetic analyses are clearly pointing at a joint activity of both loci to control the onset of bolting.

### The floral promoter *BvFT2* is completely downregulated in beets which do not bolt after vernalization

**Figure 2:**
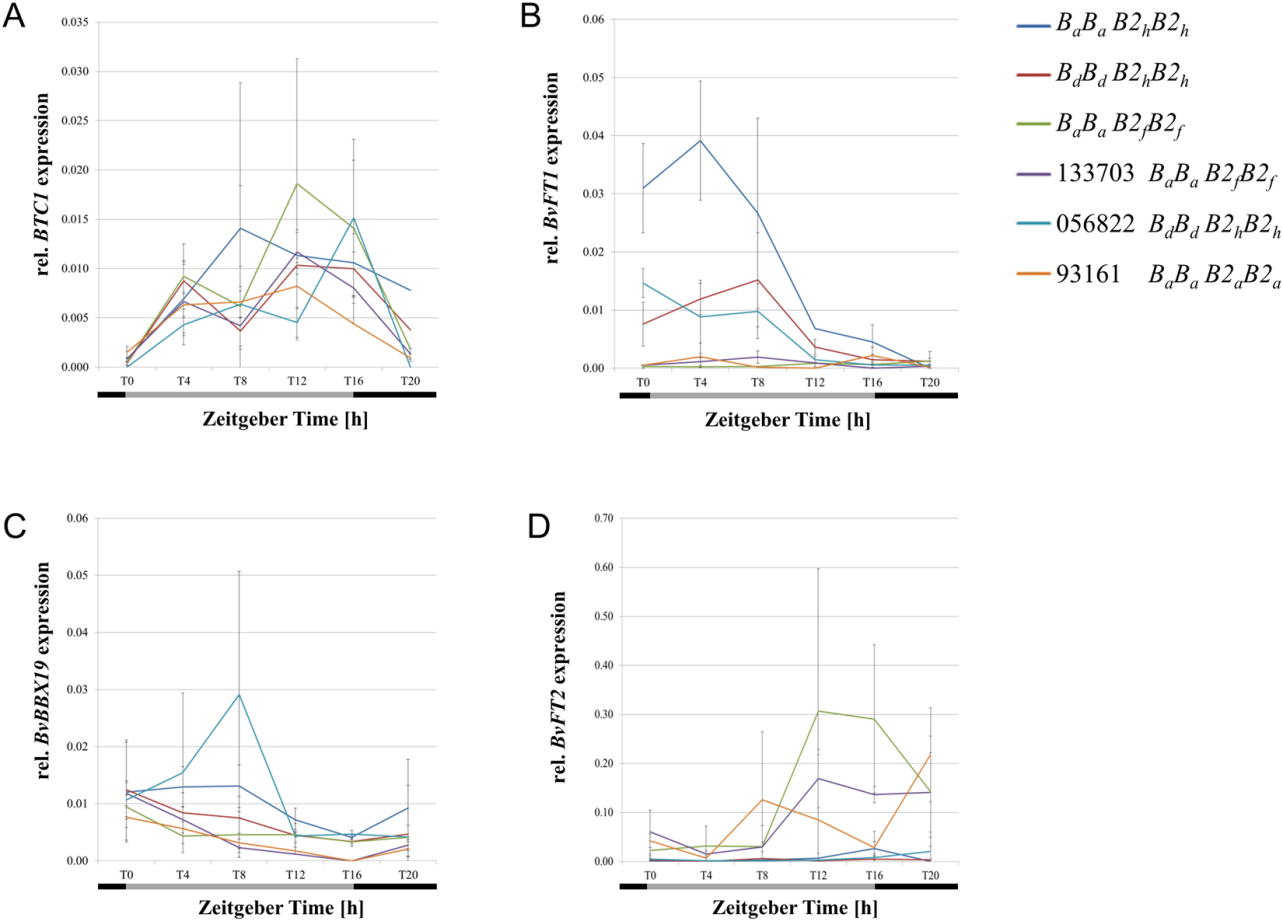
Diurnal expression analysis for *BTC1*, *BvBBX19*, *BvFT1* and *BvFT2* in bolting and non-bolting F_2_ plants after cold treatment. The expression was measured in bolting (*B*_*a*_*B*_*a*_ *B2*_*f*_*B2*_*f*_) or non-bolting (*B*_*a*_*B*_*a*_ *B2*_*h*_*B2*_*h*_, *B*_*d*_*B*_*d*_ *B2*_*h*_*B2*_*h*_) F_2_ plants after vernalization. The *BvBBX19* mutant parent 056822 (*B*_*d*_*B*_*d*_ *B2*_*h*_*B2*_*h*_) and the biennial genotypes 133703 (*B*_*a*_*B*_*a*_ *B2*_*f*_*B2*_*f*_) and 930176 (*B*_*a*_*B*_*a*_ *B2*_*a*_*B2*_*a*_) were used as controls. Each value is the mean of three biological and three technical replicates, except for the *BvFT1* expression of the *B*_*a*_*B*_*a*_*B2*_*h*_*B2*_*h*_ genotype where each value is the mean of two biological replicates and three technical replicates. The relative gene expression is given on the vertical axis. Night and day periods are indicated by black and grey bars. Error bars represent the SD of biological replicates.

We questioned whether the transcript levels of *BTC1* and *BvBBX19* differ between F_2_ plants bolting and non-bolting after vernalization. Therefore, the effect of different *B*_*a*_*/B*_*d*_ and *B2*_*f*_/*B2*_*h*_ allele combinations on their transcriptional activity was investigated using the same F_2_ plants as for the genetic experiments. Leaves were taken 23 days after cold treatment every 4 hours over 24 hours. We observed no significant differences in the diurnal expression pattern of *BTC1* between double homozygous F_2_ plants (*B*_*a*_*B*_*a*_ *B2*_*f*_*B2*_*f*_), F_2_ plants that are homozygous for the *B2*_*h*_ allele and the biennial controls (Fig. 2A) despite of strikingly different life cycle regimes. *BTC1* was upregulated during the day and the transcript levels decreased during the night in controls as well as in F_2_ individuals bolting after vernalization (*B*_*a*_*B*_*a*_ *B2*_*f*_*B2*_*f*_) and F_2_ genotypes non-bolting after vernalization (*B*_*d*_*B*_*d*_ *B2*_*h*_*B2*_*h*_ and *B*_*a*_*B*_*a*_ *B2*_*h*_*B2*_*h*_). For *BvBBX19* we detected generally low expression levels during the day with continuously increasing transcript levels during the night in all plants which bolted after vernalization except for the parental genotype 056822 (*B*_*d*_*B*_*d*_ *B2*_*h*_*B2*_*h*_). In general, the *BvBBX19* transcript levels were increased during the day (ZT8) in all plants which is in accordance with previous data^8^. Interestingly, upregulation was also observed in F_2_ genotypes that failed to bolt after the cold treatment (*B*_*d*_*B*_*d*_ *B2*_*h*_*B2*_*h*_ and *B*_*a*_*B*_*a*_ *B2*_*h*_*B2*_*h*_) however at a much lower level when compared to the parental genotype 056822 (Fig. 2C).

We reasoned that the *BTC1/BvBBX19* genotype impacts the expression of the two *FT* paralogs *BvFT1* and *BvFT2*. It had been demonstrated that floral transition in beet is promoted through downregulation of the floral repressor *BvFT1* and therewith upregulation of the floral inducer *BvFT2*, which both are downstream targets of *BTC1* and *BvBBX19*^5,6,8^. We observed that the transcriptional activity of *BvFT1* and *BvFT2* follows the anticipated expression pattern (Fig. 2B,D). As expected, *BvFT2* was highly upregulated and *BvFT1* completely downregulated after vernalization in the biennial controls and in biennial F_2_ plants. Interestingly, a contrasting expression pattern was observed in F_2_ plants which did not bolt after vernalization. The transcriptional activity of *BvFT1* was two times higher in non-bolting F_2_ plants homozygous for *B*_*a*_*/B2*_*h*_ as compared to F_2_ plants homozygous for *B*_*d*_/*B2*_*h*_.

### BvBBX19 and BTC1 physically interact with each other

**Figure 3:**
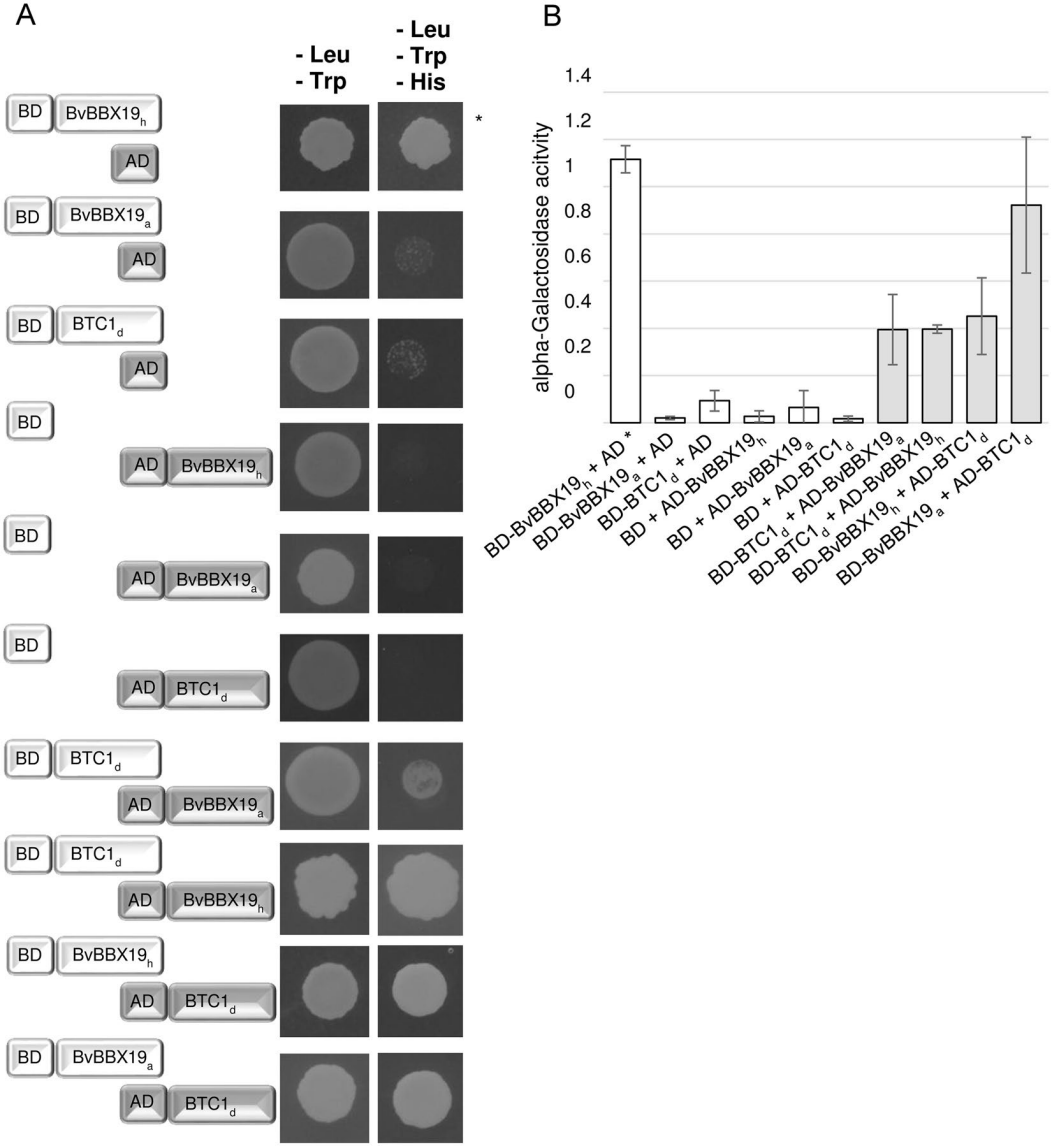
Yeast-2-Hybrid analysis showing interaction of BTC1 with BvBBX19. The proteins were fused at their N-terminus to either the DNA binding domain (BD) or the activation domain (AD) of the *GAL4* transcription factor. (**A**) Yeast cells were transformed with vectors harboring the indicated constructs. Aliquots of overnight cultures were spotted on non-selective (-Leu, -Trp) or selective (-Leu, -Trp, -His) plates and tested for His auxotrophy. Nine clones of each plasmid combination were tested and one representative result is shown. (**B**) Quantification of BvBBX19/BTC1 interaction using α-galactosidase assay. Means ± SD of three technical replicates are displayed. The BvBBX19 mutant (BvBBX19_h_) with a premature stop codon after the first B-Box domain was included in these studies. BvBBX19_h_ showed autoactivation (indicated by asterisks).

The absence of a gene in sugar beet encoding a canonical CO protein suggested that one or several other proteins fulfill the function of CO in this plant. The most likely candidates are BvBBX19 and BTC1 since they contain two B-Box domains and the CCT domain, respectively resembling the CO domain structure^8^. One likely scenario how BvBBX19 together with BTC1 can replace CO is direct physical interaction between the two proteins resulting in a functional CO ortholog. To test this hypothesis, we performed yeast-two-hybrid studies. Constructs were made containing the full-length coding regions of *BvBBX19* and *BTC1* fused to either the GAL4 DNA-binding domain (BD) or the GAL4 activation domain (AD) at the N-terminus of the respective proteins. In addition, we included constructs of the previously identified BvBBX19 mutant (BvBBX19_h_), which contains a premature stop codon resulting in a BvBBX19 variant with only one B-Box^8^. Again, AD or BD-domains were fused to the N-terminus of BvBBX19_h_. Wild type BvBBX19_a_ and BTC1_d_ constructs showed no autoactivation and were thus useful to study interaction between the two proteins. For both combinations of wild type BvBBX19_a_with BTC1_d_ we observed growth of yeast cells on selective plates (-Leu, -Trp, -His) as well as induction of the α-galactosidase reporter in the quantitative assays (Fig. 3). Thus, BvBBX19_a_ and BTC1_d_ interact. In case of BvBBX19_h_ we observed autoactivation for the BD-BvBBX19_h_ construct. Thus, this construct was not useful for further interaction studies. However, AD-BvBBX19_h_ did not result in autoactivation. In combination with BD-BTC1_d_, colonies were formed on selective medium and α-galactosidase activity induced. This result implies that the second C-terminally located B-box in BvBBX19_a_, which is missing in BvBBX19_h_, is not essential but supportive for the interaction with BTC1_d_.

**Figure 4:**
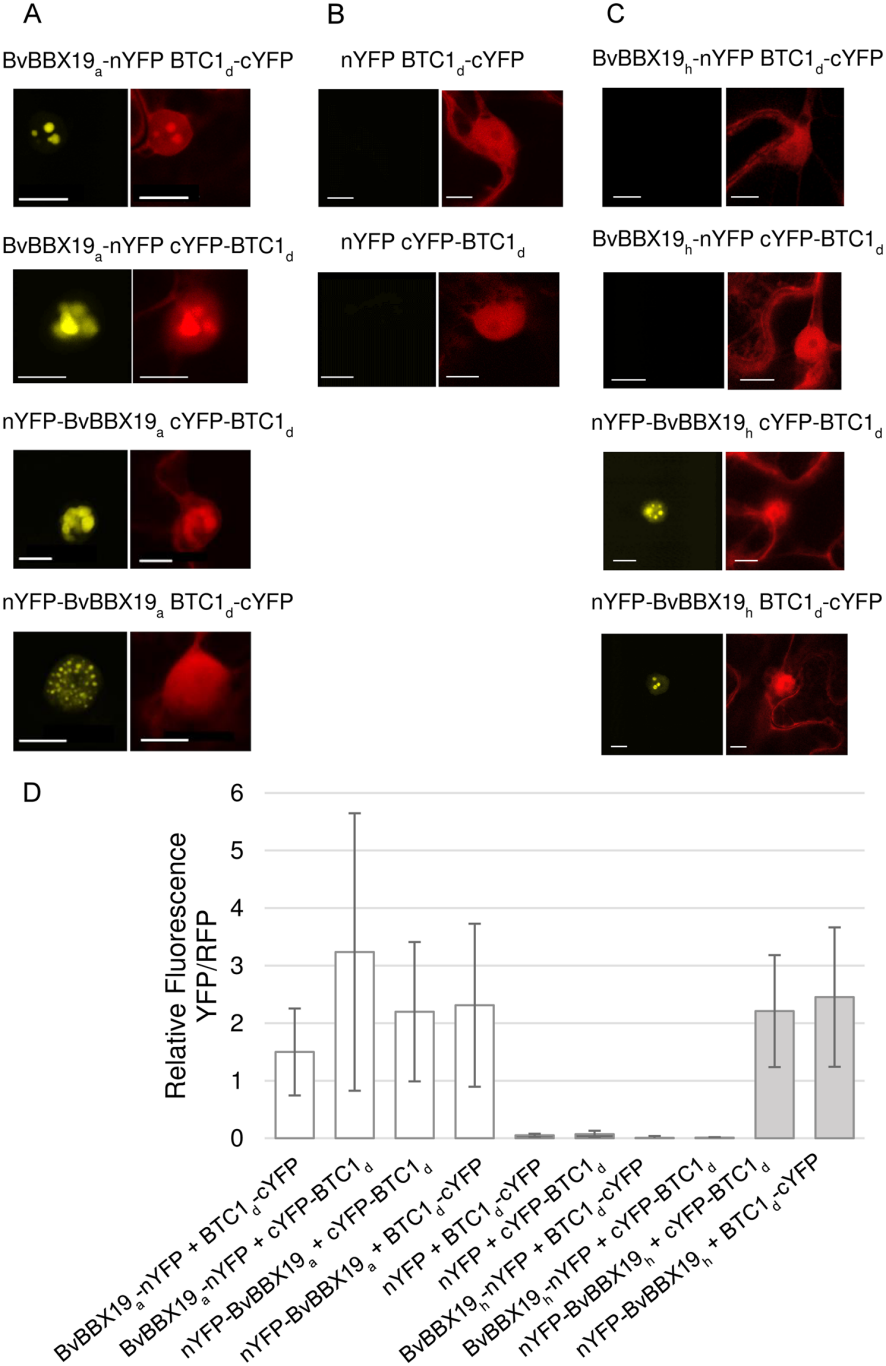
Interaction between BvBBX19 and BTC1 analyzed by ratiometric bimolecular fluorescence complementation (rBiFC). (**A–C**) Confocal pictures of *Nicotiana benthamiana* leaves three days after *Agrobacterium tumefaciens* infiltration with the rBiFC constructs. The C-terminal part of YFP (cYFP) or the N-terminal part of YFP (nYFP) was fused to the target proteins (X) at their N-terminus (c/nYFP-X) or at their C-terminus (X-c/nYFP). Fluorescence was detected with a Leica TCS SP5 Confocal Laser Scanning Microscope. YFP was excited with a 488 nm laser and RFP with a 561 nm laser. YFP fluorescence was detected between 535 nm and 560 nm. RFP fluorescence was detected between 600 nm and 625 nm. (**A**) shows BvBBX19_a_ interaction with BTC1_d_, (**B**) negative controls for BTC1_d_, and (**C**) interaction of mutant BvBBX19 (BvBBX19_h_) with BTC1_d_. Scale bars, 10 µm. (**D**) Quantification of the mean fluorescence of split-YFP normalized against RFP. Data are means ± SD of at least 20 images selected at random.

Y2H data strongly suggested direct physical interaction between BvBBX19 and BTC1. For further confirmation, we applied ratiometric bimolecular fluorescence complementation assays (rBiFC). We used constructs where either the 5′- or the 3′ region of BvBBX19_a_ was fused with the 5′-terminal half of YFP (nYFP). Accordingly, BTC1_d_ was fused with the C-terminal part of YFP (cYFP) at its N- or C-terminus. These constructs were co-transfected into *Nicotiana benthamiana* leaves using Agrobacterium-mediated infiltration. All four combinations resulted in YFP signals (Fig. 4A) in contrast to co-expression of the non-fused nYFP controls with BTC1_d_ fused to cYFP at its N-terminus or C-terminus (Fig. 4B). The truncated BBX19 version nYFP-BvBBX19_h_ co-transfected with BTC1_d_ carrying cYFP at the N-terminus or C-terminus also gave a clear YFP signal in contrast to BvBBX19_h_ constructs carrying nYFP at the C-terminus of BvBBX19_h_ (Fig. 4C). The latter result is expected since BvBBX19_h_ contains a stop codon upstream of the second B-Box and thus does not allow expression of the C-terminal YFP half. Interestingly, in all cases complemented YFP signals were observed in nuclear bodies. Quantification of the YFP against the RFP fluorescence signals from at least 20 images (Fig. 4D) are consistent with the representative pictures presented in Fig. 4A–C.

## Discussion

We have performed a genetic study with all combinations of *BTC1* (*B*) and *BvBBX19* (*B2*) alleles in an F_2_ population. *B*_*d*_ is a typical annual allele only found in wild beet populations from the Mediterranean. *B*_*a*_ carries six non-synonymous SNPs and a large insertion in the promoter compared to ‘annual’ alleles^6^. *B2*_*f*_ is found in annual beets^9^ while *B2*_*h*_ is a nonsense EMS mutant allele^8^.

Beet is a typical long day plant. Bolting even in the presence of the early bolting alleles or after vernalization is strongly delayed in short days^19^. Therefore, all experiments were performed under long day conditions. An annual life cycle requires an annual *B* allele and a functional *B2* allele (*B*_*d*_/*B2*_*f*_). The competence for early flowering is lost in plants homozygous for the *B2* mutant allele *BvBBX19*_*h*_ irrespective of the *B* allele. Likewise, *B* knockdown (RNAi) plants cannot flower any more even after vernalization^6^.

Double mutants homozygous for the biennial *B* and the non-functional *B2* allele completely lost their competence to bolt which confirmed our initial hypothesis that *B* and *B2* jointly regulate the onset of bolting in sugar beet. By combining two mutant alleles, we could select plants that did not bolt even after cold treatment. We found an incomplete epistatic interaction between bolting resistant plants among all *B2*_*h*_ homozygous genotypes, but the presence of the *B*_*d*_ alleles modified the *B2*_*h*_ effect because biennial plants were present in the *B*_*d*_*B2*_*h*_ parent and F_2_ plants. We assume that apart from these two major bolting time regulators, additional genes can modify bolting time. Moreover, the rare occurrence of spontaneously bolting plants in production fields points at environmental factors modifying the activities of *B* and *B2*. These factors together may explain the presence of biennial plants in the 056822 parent.

The genus *Beta* comprises iteroparous perennials with an annually repeated requirement for vernalization^20^. Future studies with perennial wild beets will resolve the question whether the *BTC1*/*BvBBX19* module and the *BR1* QTL^4^ control the perennial life cycle. However, strictly non-bolting genotypes are likely to be a dead end of evolution because they cannot reproduce sexually in contrast to iteroparous plants which flower and set seeds in subsequent years after winter. Thus, it is no surprise that despite of extensive screenings *BTC1*/*BvBBX19* double mutants have not been found in nature so far.

The *B2*_*h*_ genotypes exhibited a strong requirement for vernalization even in the presence of the early bolting allele *B*_*d*_. This indicates that there are upstream regulators of the *BTC1*/*BvBBX19* module which respond to cold temperatures and to alterations of the *B2*_*h*_ protein. This makes *B2* a primary target of a putative vernalization regulatory pathway. However, no further mutants have been detected so far. Searching for orthologous genes from Arabidopsis has not been successful and beet lacks a functional ortholog of *FLOWERING LOCUS C* which is a major integrator of signals from the vernalization pathway in Arabidopsis. Seemingly, divergent vernalization pathways have evolved in both species. Because vernalization has an epigenetic basis, genes responding to methylation might be interesting candidates. Consequently, two genes, *SHORT VEGETATIVE PHASE* (*BvSVP*) and *BvVIN3* come into focus as upstream regulators of the *BTC1/BvBBX19* module because they are hypomethylated and/or differentially expressed after cold exposure^21,22^.

How can the bolting-resistant phenotype be explained by protein-interaction and expression studies? *BTC1* requires a functional *BvBBX19* protein consistent with our data that beets carrying the annual *B*_*d*_ allele do not bolt in the presence of the mutant *B2*_*h*_ allele. Loss of competence to bolt is due to downregulation of the floral inducer *BvFT2* and upregulation of the floral repressor *BvFT1*^6^. The non-bolting phenotype of the *B2* mutant is not caused by a lack of protein-interaction because binding between BvBBX19_h_ and BTC1_d_ was demonstrated (Figs 3 and 4). Y2H data imply that BBX19 and BTC1 do not require another beet protein for their interaction. However, it cannot be excluded that BTC1 and BvBBX19 interact with other proteins as CO does with PHYTOCHROME INTERACTING FACTOR 4^23^. We propose a model where *BTC1* and *BvBBX19* (mutated and wild type) dimerize to bind to the *BvFT2* promoter. Likewise, the interaction between Arabidopsis COL proteins and CO has been demonstrated. B-BOX 32 binds to CONSTANS-LIKE3^15^ and BBX19 binds to CO to suppress its function as an activator of *FT*^17^. Consequently, the heterodimer cannot bind to the Box 1 Motif of the *FT* promoter which is essential for binding of the CO protein. We reason that BTC1 or BvBBX19 alone are not able to bind to the *BvFT2* promoter and that the heterodimer of mutant BvBBX19_h_ with the BTC1_d_ protein cannot bind to the *BvFT2* promoter. The importance of intact domains for their binding activities was recently reported for Arabidopsis where a truncated COβ variant lacking the CCT domain lost its DNA-binding affinity^24^. The variant protein results from alternative splicing of the CO mRNA. Moreover, the truncated protein inhibits the function of the full-size COα protein by reducing its protein abundance and preventing its DNA-binding. A similar mechanism of CO-BBX functional interaction has been reported for rice where *OsBBX14* activates the CO ortholog *Hd1* which is a repressor of the rice *FT* ortholog *Heading date* 3a (*Hd3a*) under LD conditions^25^. In Arabidopsis, CO was shown to bind to a tandemly repeated sequence element of the *FT* promoter [consensus TGTG(N2-3)ATG motif]^26^. A promoter analysis of the beet *FT* genes revealed that this element is lacking from the 5′ regions of *BvFT1* and *BvFT2* (Supplementary Table 5). Moreover, overexpression of *BvBBX19* and *BTC1* in Arabidopsis CO mutants did not accelerate flowering (data not shown). Evidently, plants have evolved different mechanisms to control *FT* expression. In Arabidopsis and rice, *CO-*like genes and *CO* orthologs gained different functions as both activators and suppressors of their downstream target. We propose an alternative mechanism for beet, where two *FT* paralogs are differentially regulated by two *CO-*like genes whose function depends on vernalization. The upstream regulators responding to external cues are still unknown. Moreover, the involvement of other homologs of CO binding proteins such as *TARGET OF EAT1* (*TOE1*) or *small B-BOX protein* (*MiP1a* and *MiP1b*)^11^ from Arabidopsis remains to be demonstrated.

Simon *et al*.^27^ suggested, that *CO* has been derived from *COL* genes and that the function of the CO protein, which is specific to Brassicaceae species gave Arabidopsis an adaptive advantage during its expansion to northern geographical regions. Also *B. maritima* spread to northern regions after the last ice age. It is tempting to speculate that the flexible *B/B2* module was an important factor for its adoption to winter climates and LD conditions. We reason that *BTC1* and *BvBBX19* must also perceive signals from the photoperiod and the vernalization signaling pathways because bolting initiation depends on long days and exposure to cold temperatures. A recent study with Arabidopsis demonstrated that the PRR proteins play an important role in stabilizing the CO protein. They suppress the proteasomal degradation of CO and contribute to light-mediated accumulation of CO during the day^28^. In beet, one member of the PRR clade has been further studied. Interestingly, *BvPRR7* is a cold responsive gene with a clock function and caused late flowering after overexpression in Arabidopsis^7^. Future studies are needed to show if this gene plays a role in beet as an upstream regulator of the *BTC1/BvBBX19* module.

This study has importance for breeding vegetative crops which are sown in spring under winter climate conditions. After early sowing, cold temperatures can pose a risk because they cause early bolting which drastically reduces yield (e.g. cabbage, carrots, salad, beet root)^29^. Therefore, breeders have been selecting for bolting resistant mutants, many of these carry mutations in functional orthologs of *FLC* (only Brassica species), *CO* or *FT*. Breeding winter beets requires full bolting control after winter. In contrast to traditional ‘spring beets’, they must not bolt after winter. This can be achieved by selecting for non-bolting (after vernalization) alleles from the *BR1* QTL on chromosome 9^4,30^. Alternatively, we propose a haplotype-based breeding strategy using well defined *BTC1* and *BvBBX19* alleles. But how can we harvest seeds from the parents if they are already bolting resistant? This problem could be overcome by introducing the early bolting *B* allele (e.g. *B*_*d*_) into non-bolting parents turning them into biennials which can flower and set seeds after winter. We propose a haplotype swapping strategy where different *B* and *B2* alleles^9^ are combined with each other. A second approach relies on conditional bolting of *B*_*h*_ parents. Non-bolting plants can enter the reproductive phase under extreme environments. We have obtained seeds from *B*_*h*_ genotypes after cultivation in a climate chamber under 24 hours light and largely extended vernalization period. As an alternative, the bolting resistance of *B*_*h*_ seed parents could be overcome by field cultivation in southern regions under high temperatures. It was recently shown, that *CO* expression increases under high temperatures^23^. Although this was observed under SD conditions, it is tempting to speculate that *B*/*B2* allele combinations display different temperature sensitivity before and after vernalization.

## Materials and Methods

### Plant material and growth conditions

We performed a cross between two single plants of the biennial beet lines seed code 056822 (plant #15) and 093187 (plant #8). The female parent 056822 carries the *BTC1*_*d*_ allele only found in annual beets which confers early bolting without vernalization^6^ and a mutated *BvBBX19* allele^8^ which we termed *BvBBX19*_*h*_ following the haplotype nomenclature described by Höft *et al*.^9^. The pollinator parent 093187/8 carries the *btc1*_*a*_ allele and the functional *BvBBX19*_*f*_ allele. For ease of understanding we will use the allele nomenclature as *B*_*d*_ (haplotype *BTC1*_*d*_), *B*_*a*_ (haplotype *btc1*_*a*_), *B2*_*f*_ (haplotype *BvBBX19*_*f*_) and *B2*_*h*_ (haplotype *BvBBX19*_*h*_). A single F_1_ plant (seed code 133580/1, *B*_*d*_*B*_*a*_ *B2*_*h*_*B2*_*f*_) was selected and propagated by bag isolation to produce F_2_ seeds (seed code 142063).

For phenotyping, 145 plants of the F_2_ population 142063 were grown in a climate chamber under long day conditions (16 h light/8h dark, 320 µmol m^−2^ s^−1^) for 325 days. The two parent lines 056822 and 133703 (selfing progeny of 093187), three biennial (seed codes 092492, 930184, 930176) and two annual genotypes (001684, 991971) were grown as controls (five plants per line). Plants were first grown in 9 cm pots for 135 days at 20 °C and then cold treated at 4 °C for 12 weeks, followed by an acclimatization phase at 12 °C for three days. For the rest of the experiment, they grew again in 11 cm pots at 20 °C for another 102 days. Every second day, plants were randomized and the onset of bolting was recorded (BBCH scale code: 51) according to Meier *et al*.^31^. Finally, plants were classified as follows: (1) annual plants which bolted within 135 days, (2) biennial plants which bolted only after cold treatment, and (3) plants, which did not bolt until the end of the experiment after 325 days.

### DNA techniques

For DNA isolation, leaves were harvested from six-weeks-old F_2_ plants and freeze dried. Genomic DNA was isolated applying the CTAB method^32^. A 10-fold dilution of the extracted DNA was later used for PCR using Taq DNA Polymerase (Invitrogen). We used the InDel marker CAU4234 and the CAPS marker CAU4235 for genotyping the *BTC1* and *BvBBX19* locus, respectively (Supplementary Table 2). PCR products were separated on 1% agarose gels.

### Gene expression analysis

We measured the diurnal expression of the four flowering time genes *BTC1*, *BvBBX19*, *BvFT1* and *BvFT2* by qRT-PCR in F_2_ plants with the *BTC1* and *BvBBX19* haplotypes *B*_*a*_*B*_*a*_ *B2*_*f*_*B2*_*f*_, *B*_*a*_*B*_*a*_ *B2*_*h*_*B2*_*h*_ and *B*_*d*_*B*_*d*_*.B2*_*h*_*B2*_*h*_, and in the biennial controls 056822 (*B*_*d*_*B*_*d*_ *B2*_*h*_*B2*_*h*_), 133703 (*B*_*a*_*B*_*a*_ *B2*_*f*_*B2*_*f*_) and 930176 (*B*_*a*_*B*_*a*_ *B2*_*a*_*B2*_*a*_). Total RNA was isolated from young leaves that were harvested 23 days after cold treatment in a 4 hours interval over 24 hours (first measurement at ZT 0, the time of lights on). Total RNA was extracted with the peqGOLD Plant RNA Kit (PeqLab) and subsequently treated with DNase. 500 ng of total RNA was reverse transcribed using a First Strand cDNA Synthesis kit (Fermentas). Resulting cDNA was diluted 10-fold and 2 µl of the dilution were used as a template for qRT-PCR. Three independent biological and three technical replicates were analyzed. qRT-PCR was performed with a Platinum SYBR Green Mastermix (Invitrogen) on a CFX96 Real-Time PCR detection system (Bio-Rad) with a final reaction volume of 20 µl and a final primer concentration of 20 pM. The housekeeping gene *BvGAPDH* was used as a reference. Data were analyzed with the CFX Manager Software v2.1 (Bio-Rad). Expression levels were first calculated with the comparative CT (Δ_CT_) method and then normalized to the geometric mean of *BvGAPDH* to calculate the relative expression levels.

### Yeast-2-Hybrid assays

Yeast-2-Hybrid experiments were performed using the Matchmaker Gold Yeast-Two-Hybrid System (Clontech). The proteins of interest were fused at their N-terminus to either the DNA binding domain (BD) or the activation domain (AD) of the *GAL4* transcription factor by insertion of the full-length coding sequences of *BvBBX19*_*a*_, *BvBBX19*_*h*_ (*BvBBX19* mutant) and *BTC1*_*d*_ into the *Nco*I and *Xho*I sites of the vector pACT2 or the *Nco*I and *Sal*I sites of the vector pAS2-1. Full-length coding sequences of *BvBBX19*_*a*_, *BvBBX19*_*h*_ and *BTC1*_*d*_ were obtained by PCR with primers listed in Supplementary Table 2. The correctness of the amplified sequences was verified by sequencing. Yeast cells (strain Y2H Gold, Clontech) were transformed according to the supplier’s manual. Screening for histidine auxotrophy was done with nine clones of each transformant which were spread on non-selective (-Leu, -Trp) or selective (-Leu, -Trp, -His) plates and incubated for two days at 28 °C.

Quantification of interaction was determined by the α-galactosidase-assay^33^ with minor modifications. For this purpose, yeast transformants were cultured overnight in 3 mL selective medium. After measuring OD_600_ of the overnight culture and pelleting the cells, 200 µL of the overnight medium were mixed with 600 µL assay buffer (0.33 M sodium acetate, pH 4.5, 10 mg mL^−1^ p-nitrophenyl-alpha-D-galactopyranoside) and incubated at 29 °C. After 21 h of incubation 200 µL stopping buffer (2 M sodium carbonate) were added and OD_410_ was measured. α-galactosidase activity was calculated as: α-galactosidase units = 1,000 × OD_410_/(t × V × OD_600_), where t = time of incubation in min, V = volume of culture, OD_410_ = absorbance by p-nitrophenol, OD_600_ = cell density at the beginning.

### Ratiometric Bimolecular Fluorescence Complementation (rBiFC) and immunoblots

For rBiFC, *BvBBX19*_*a*_, *BvBBX19*_*h*_ and *BTC1*_*d*_ were C- or N-terminally fused to either the N-terminal or the C-terminal half of YFP (n/cYFP). Thus, eight different construct combinations were obtained and the unfused N-terminal half of YFP was used as negative control in combination with cYFP-BTC1_d_ or BTC1_d_ -cYFP.

For generation of constructs, the full-length coding sequences of *BvBBX19*_*a*_, *BvBBX19*_*h*_ and *BTC1*_*d*_ were PCR amplified with *att* sites allowing recombination into the entry vectors pDONR221-P1P4 and pDONR221-P3P2 followed by recombination into the Gateway vector pBiFCt-2in1 that also provides RFP as an internal standard^34^. The obtained constructs were transformed into *Agrobacterium tumefaciens* strain GV3101(pMP90)^35^. 4-weeks-old *Nicotiana benthamiana* plants were transiently co-transformed by Agrobacterium infiltration^36^ with the construct combinations mentioned above and with p19 to suppress gene silencing^37^. Three days after infiltration YFP complementation was analyzed using a Leica TCS SP5 Confocal Laser Scanning Microscope (Leica). YFP was excited with a 488 nm laser and RFP with a 561 nm laser. YFP fluorescence was detected between 535 nm and 560 nm, RFP fluorescence between 600 nm and 625 nm. Quantification of the mean fluorescence of split-YFP was done by normalization against RFP. Data were calculated as means of at least 20 images selected at random. Relative fluorescence was determined using ImageJ estimating the mean grey value of the different pictures within an area of around 5 pixels. The maximum grey value per pixel of YFP fluorescence was set as 225.

Expression of *BvBBX19*_*a*_ or *BvBBX19*_*h*_ fused to nYFP or unfused nYFP (as negative control for rBiFC) was detected via the HA-tag positioned at the C-terminus of nYFP (Fig. S1). Proteins were extracted from infiltrated leaf tissues by TCA precipitation^38^. 40 µg of proteins per lane were separated by SDS-PAGE. After blotting, the nitrocellulose membrane was blocked with 7% milk powder in TBS and probed with rat anti-HA antibody (Roche, 11867423001, 1:1,500 in TBS-T) and secondary αRat-HRP antibody (Millipore, NMM1767593, 1:10,000 in TBS-T) using the ECL detection assay (Bio-Rad) according to supplier’s manual.

## Electronic supplementary material

Supplementary Data
