# Supplementary material for: Two CONSTANS-LIKE genes jointly control flowering time in beet
Source: Sci Rep. 2018 Oct 31;8:16120. doi: 10.1038/s41598-018-34328-4 (PMC6208394; doi:10.1038/s41598-018-34328-4)
Supplement: Supplementary file 1 — Supplementary Data [file 41598_2018_34328_MOESM1_ESM.docx]

Supplementary information

Two CONSTANS-LIKE genes jointly control flowering time in beet

Nadine Dally^1^, Maike Eckel^3^, Alfred Batschauer^3^, Nadine Höft^2^ and Christian Jung^2*^

^1^UKSH Campus Kiel, Hematology Laboratory Kiel, Langer Segen 8-10, D-24105 Kiel, Germany

^2^Plant Breeding Institute, Christian-Albrechts-University of Kiel, Am Botanischen Garten 1-9, D-24118 Kiel, Germany

^3^Department of Plant Physiology and Photobiology, Faculty of Biology, Philipps-University of Marburg, Karl-von-Frisch-Str. 8, D-35032 Marburg, Germany

*Corresponding author email: c.jung@plantbreeding.uni-kiel.de

Correspondence to: Prof. Dr. Christian Jung
 Plant Breeding Institute
 Christian-Albrechts-University of Kiel
 Am Botanischen Garten 1-9
 D-24118 Kiel
 Germany
 Tel.: 0431-880 7364
 Fax: 0431-880 2566

E-mail: c.jung@plantbreeding.uni-kiel.de

# Supplementary data

**Supplementary Table 1** Haplotypes of four bolting time genes used in this study and markers derived thereof.

| Gene | Allele | Phenotype | Origin | Sequence Variation/ Mutation | Putative Effect | Marker | Marker Type | Marker Position |
| --- | --- | --- | --- | --- | --- | --- | --- | --- |
| *BTC1* | *btc1_a_* (*B_a_* ) | biennial, in combination with *BvBBX19_f_* | sugar beet | reference sequence |  | CAU4234 | InDel | exon 3, nt 302 and exon 4 nt 134 |
| *BTC1* | *BTC1_d_* (*B_d_* ) | annual, in combination with *BvBBX19_f_* | sugar beet | differs from *btc1_a_* by 11 non-synonymous and 3 synonymous polymorphisms | 11 amino acid changes | CAU4234 | InDel | exon 3, nt 302 and exon 4 nt 134 |
| *BvBBX19* | *BvBBX19_f_* (*B2 _f_*) | annual, in combination with *BTC1_d_* | sugar beet | reference sequence |  | CAU4235 | CAPS, *Dra*III | intron 1, nt 420 to exon 4, nt 248 |
| *BvBBX19* | *BvBBX19_h_* (*B2_h_*) | biennial, in combination with *BTC1_d_* | EMS mutant | differs from *BvBBX19_f_* by 1 SNP in intron 2, nt 1 | stop codon resulting in truncated protein | CAU4235 | CAPS, *Dra*III | intron 1, nt 420 to exon 4, nt 248 |

Supplementary Table 2 PCR primer sequences and conditions of molecular markers used in this study and for cloning full-length coding sequences of *BTC1* and *BvBBX19* into vectors for Yeast-2-Hybrid experiments.

| Marker name | Target | Marker type | Target vector | Primer name | Primer sequence 5`-->3` | PCR conditions | Fragment size [bp] | Fragment size [bp] Seed parent (056822/15) | Fragment size [bp] Pollinator (093187/8) |
| --- | --- | --- | --- | --- | --- | --- | --- | --- | --- |
| CAU4234 | *BTC1* | InDel |  | A882^a^ | GTGACTCATTATCTTGGACAG | 94°C, 2' + [(94°, 30'' + 60°C, 30'' + 72°C, 45'') x 32] + 72°C, 5' |  | 790 | 1100 |
|  |  |  |  | A883 | AGAAAGTGATGACTCAACTCG |  |  |  |  |
| CAU 4235 | *BvBBX19* | CAPS, digestion with *Dra*III |  | N0150_F | ATGCATCTTATACTATGGGACCAC | 94°C, 2' + [(94°, 30'' + 57°C, 30'' + 72°C, 60'') x 32] + 72°C, 5' |  | 742 | 537 + 205 |
|  |  |  |  | N0150_R | GGAACAATGCTTGCCGAATCAC |  |  |  |  |
| - | *BTC1* | - | pACT2 |  | ATACCATGGCTAGGTTGATTCACAAAAATGAAG | 98°C, 30'' + [(98°, 10'' + 71°C, 30'' + 72°C, 1') x 43] + 72°C, 2' | 2384 |  |  |
|  |  |  |  |  | TATCTCGAGTTAGCTATCGGTATTCCTTCCTTTGTTTTC |  |  |  |  |
|  |  | - | pAS2-1 |  | ATACCATGGCTAGGTTGATTCACAAAAATGAAG | 98°C, 30'' + [(98°, 10'' + 70°C, 30'' + 72°C, 1') x 43] + 72°C, 2' | 2384 |  |  |
|  |  |  |  |  | TATCTCGAGTTAGCTATCGGTATTCCTTCCTTTGTTTTC |  |  |  |  |
| - | *BvBBX19* | - | pACT2 |  | ATACCATGGCTAGGACTCTTTGTGATGTTTGTG | 98°C, 30'' + [(98°, 10'' + 71°C, 30'' + 72°C, 1') x 43] + 72°C, 2' | 605 (BvBBX19_a_), 695 (BvBBx19_h_) |  |  |
|  |  |  |  |  | TATCTCGAGTCATTTTTCTGGCTCGCTTTTG |  |  |  |  |
|  |  | - | pAS2-1 |  | ATACCATGGCTAGGACTCTTTGTGATGTTTGTG | 98°C, 30'' + [(98°, 10'' + 72°C, 30'' + 72°C, 1') x 43] + 72°C, 2' | 605 (BvBBX19_a_), 695 (BvBBx19_h_) |  |  |
|  |  |  |  |  | TATGTCGACTCATTTTTCTGGCTCGCTTTTG |  |  |  |  |

Supplementary Table 3 Genotypes and phenotypes of F_2_ plants from the population 142063. Never bolting plant did not bolt until the end of the experiment (325 days after sowing). *BTC1* (*B*) and *BvBBX19* (*B2*) genotypes were determined by PCR using the markers CAU4234 and CAU4235. The respective haplotypes for *B* and *B2* are given in lowered letters.

| Genotype | F_2_ Plant | Days to bolting after sowing |
| --- | --- | --- |
| *B_a_B_a_ B2_h_B2_h_* | 142063/15 | never bolting |
|  | 142063/17 | never bolting |
|  | 142063/31 | never bolting |
|  | 142063/40 | never bolting |
|  | 142063/41 | never bolting |
|  | 142063/51 | never bolting |
|  | 142063/84 | never bolting |
|  | 142063/93 | never bolting |
|  | 142063/94 | never bolting |
|  | 142063/100 | never bolting |
|  | 142063/105 | never bolting |
|  | 142063/114 | never bolting |
|  | 142063/118 | never bolting |
|  | 142063/119 | never bolting |
|  | 142063/125 | never bolting |
|  | 142063/135 | never bolting |
|  | 142063/143 | never bolting |
| *B_d_B_d_ B2_h_B2_h_* | 142063/9 | never bolting |
|  | 142063/78 | 260 |
|  | 142063/99 | never bolting |
|  | 142063/103 | never bolting |
|  | 142063/123 | never bolting |
| *B_d_B_a_ B2_h_B2_h_* | 142063/3 | never bolting |
|  | 142063/4 | never bolting |
|  | 142063/5 | never bolting |
|  | 142063/12 | never bolting |
|  | 142063/18 | never bolting |
|  | 142063/20 | never bolting |
|  | 142063/25 | never bolting |
|  | 142063/28 | never bolting |
|  | 142063/35 | never bolting |
|  | 142063/37 | 248 |
|  | 142063/46 | never bolting |
|  | 142063/49 | never bolting |
|  | 142063/63 | never bolting |
|  | 142063/67 | never bolting |
|  | 142063/70 | never bolting |
|  | 142063/76 | never bolting |
|  | 142063/88 | never bolting |
|  | 142063/92 | never bolting |
|  | 142063/96 | never bolting |
|  | 142063/107 | never bolting |
|  | 142063/110 | never bolting |
|  | 142063/112 | 244 |
|  | 142063/121 | never bolting |
|  | 142063/136 | never bolting |
|  | 142063/147 | never bolting |
| *B_a_B_a_ B2_f_B2_f_* | 142063/61 | 239 |
|  | 142063/72 | 237 |
|  | 142063/90 | 234 |
|  | 142063/98 | 244 |
|  | 142063/144 | 241 |
| *B_d_B_d_ B2_f_B2_f_* | 142063/1 | 36 |
|  | 142063/10 | 36 |
|  | 142063/23 | 38 |
|  | 142063/26 | 30 |
|  | 142063/36 | 38 |
|  | 142063/89 | 34 |
|  | 142063/117 | 30 |
|  | 142063/149 | 26 |
| *B_d_B_a_ B2_f_B2_f_* | 142063/11 | 93 |
|  | 142063/30 | 36 |
|  | 142063/45 | 36 |
|  | 142063/53 | 50 |
|  | 142063/62 | 69 |
|  | 142063/65 | 76 |
|  | 142063/77 | 38 |
|  | 142063/80 | 41 |
|  | 142063/82 | 34 |
|  | 142063/83 | 41 |
|  | 142063/91 | 41 |
|  | 142063/102 | 34 |
|  | 142063/120 | 38 |
|  | 142063/133 | 41 |
|  | 142063/142 | 36 |
|  | 142063/146 | 48 |
| *B_a_B_a_ B2_f_B2_h_* | 142063/8 | 244 |
|  | 142063/13 | 237 |
|  | 142063/42 | 237 |
|  | 142063/47 | 241 |
|  | 142063/48 | 239 |
|  | 142063/52 | 241 |
|  | 142063/54 | 241 |
|  | 142063/57 | 239 |
|  | 142063/60 | 239 |
|  | 142063/86 | 239 |
|  | 142063/132 | 239 |
|  | 142063/148 | 241 |
| *B_d_B_d_ B2_f_B2_h_* | 142063/6 | 29 |
|  | 142063/16 | 29 |
|  | 142063/34 | 36 |
|  | 142063/39 | 36 |
|  | 142063/50 | 55 |
|  | 142063/66 | 38 |
|  | 142063/73 | 57 |
|  | 142063/74 | 48 |
|  | 142063/81 | 29 |
|  | 142063/95 | 48 |
|  | 142063/101 | 29 |
|  | 142063/106 | 50 |
|  | 142063/113 | 41 |
|  | 142063/128 | 76 |
|  | 142063/130 | 41 |
|  | 142063/131 | 36 |
|  | 142063/138 | 69 |
|  | 142063/141 | 36 |
|  | 142063/150 | 36 |
| *B_d_B_a_ B2_f_B2_h_* | 142063/7 | 104 |
|  | 142063/19 | 43 |
|  | 142063/22 | 66 |
|  | 142063/24 | 232 |
|  | 142063/29 | 50 |
|  | 142063/32 | 232 |
|  | 142063/33 | 234 |
|  | 142063/38 | 83 |
|  | 142063/43 | 69 |
|  | 142063/44 | 90 |
|  | 142063/55 | 237 |
|  | 142063/56 | 48 |
|  | 142063/58 | 64 |
|  | 142063/59 | 83 |
|  | 142063/64 | 41 |
|  | 142063/68 | 57 |
|  | 142063/69 | 48 |
|  | 142063/71 | 79 |
|  | 142063/75 | 114 |
|  | 142063/85 | 50 |
|  |  |  |
|  | 142063/87 | 50 |
|  | 142063/97 | 73 |
|  | 142063/104 | 232 |
|  | 142063/108 | 66 |
|  | 142063/109 | 97 |
|  | 142063/111 | 43 |
|  | 142063/115 | 62 |
|  | 142063/116 | 83 |
|  | 142063/122 | 62 |
|  | 142063/124 | 79 |
|  | 142063/126 | 59 |
|  | 142063/127 | 69 |
|  | 142063/129 | 62 |
|  | 142063/134 | never bolting |
|  | 142063/137 | 239 |
|  | 142063/139 | 50 |
|  | 142063/140 | 90 |
|  | 142063/145 | 41 |

**Supplementary Table 4** Genotypic and phenotypic segregation of the F_2_ population. Plants were grown in a climate chamber under LD conditions with (biennial, never bolting) or without (annual) vernalization. Plants were classified as annuals if they bolted within 135 days after sowing. Biennials bolted within 42 days after cold treatment, never bolting plants did not bolt until the end of the experiment (102 days after cold). *BTC1* (*B*) and *BvBBX19* (*B2*) genotypes were determined by PCR using the markers CAU4234 and CAU4235. The respective haplotypes are given according to ^8^. χ^2^-tests for genotypic and phenotypic segregation were performed, the latter one for two hypotheses explaining the gene action, a dominant x dominant (dxd) and a recessive epistasis (ep) hypothesis with *B2* acting epistatically over *B*.

| F_2_ genotypes | Genotype | | Phenotype | | | | | | | | |
| --- | --- | --- | --- | --- | --- | --- | --- | --- | --- | --- | --- |
|  | expected | observed | Annual | | | Biennial | | | Never bolting | | |
|  |  |  | observed | expected  dxd | expected ep | observed | expected  dxd | expected  ep | observed | expected, dxd | expected, ep |
| *B_d_B_d_ B2_f_B2_f_* | 9.1 | 8 | 8 | 8.0 | 8.0 | 0 | 0 | 0 | 0 | 0 | 0 |
| *B_d_B_d_ B2_f_B2_h_* | 18.1 | 19 | 19 | 19.0 | 19.0 | 0 | 0 | 0 | 0 | 0 | 0 |
| *B_d_B_a_ B2_f_B2_f_* | 18.1 | 16 | 16 | 16.0 | 16.0 | 0 | 0 | 0 | 0 | 0 | 0 |
| *B_d_B_a_ B2_f_B2_h_* | 36.3 | 38 | 32 | 38.0 | 38.0 | 6 | 0 | 0 | 0 | 0 | 0 |
| *B_d_B_a_ B2_h_B2_h_* | 18.1 | 25 | 0 | 0 | 0 | 2 | 25.0 | 0 | 23 | 0 | 25.0 |
| *B_a_B_a_ B2_f_B2_h_* | 18.1 | 12 | 0 | 0 | 0 | 12 | 12.0 | 12.0 | 0 | 0 | 0 |
| *B_d_B_d_ B2_h_B2_h_* | 9.1 | 5 | 0 | 0 | 0 | 1 | 5.0 | 0 | 4 | 0 | 5.0 |
| *B_a_B_a_ B2_f_B2_f_* | 9.1 | 5 | 0 | 0 | 0 | 5 | 5.0 | 5.0 | 0 | 0 | 0 |
| *B_a_B_a_ B2_h_B2_h_* | 9.1 | 17 | 0 | 0 | 0 | 0 | 0 | 0 | 17 | 17.0 | 17.0 |
| total | 145.0 | 145 | 75 | 81.0 | 81.0 | 26 | 47.0 | 17.0 | 44 | 17.0 | 47.0 |
| χ² | 15.77^a^ | |  |  |  |  |  |  |  | 150.03^b^** | 2.24^c^ |

*α=0.05 ** α=0.01

^a^ H_0_, null hypothesis for digenic segregation, 1:2:1:2:4:2:1:2:1

^b^ H_0_, null hypothesis for segregation ratio, 9:6:1

^c^ H_0_, null hypothesis for segregation ratio, 9:4:3

**Supplementary Table 5** Bioinformatic analysis of *BvFT1* and *BvFT2* promoters using the online tool PLACE^1^. Besides the promoter region, 3500bp upstream of 5`-UTR were searched for protein binding sequences from the Arabidopsis *FT* promoter.

| **Promoter region** | **Number of elements present in** | |
| --- | --- | --- |
|  | ***BvFT1*** | ***BvFT2*** |
| CCAATBOX1 | 9 | 18 |
| MYCCONSENSUSAT | 26 | 38 |
| RAV1BAT | 1 | 0 |
| DOFCOREZM | 47 | 45 |
| CBFHV | 4 | 3 |
| DRECRTCOREAT | 2 | 0 |
| IBOXCORE | 7 | 11 |
| SORLIP1AT | 2 | 2 |
| -300CORE | 2 | 0 |
| IBOXCORENT | 1 | 0 |
| CRTDREHVCBF2 | 2 | 0 |
| LTRE1HVBLT49 | 0 | 1 |
| GT1MOTIFPSRBCS | 0 | 1 |
| TGTG(N2-3)ATG | 0 | 0 |
| **3500 bp upstream of 5`-UTR** |  |  |
| GTGANTG10 | 1 | 0 |
| POLLEN1LELAT52 | 1 | 0 |
| IBOX | 1 | 0 |
| TAAAGSTKST1 | 1 | 0 |
| NODCON1GM | 1 | 0 |
| OSE1ROOTNODULE | 1 | 0 |
| ARR1AT | 1 | 0 |
| MYBST1 | 1 | 0 |
| SP8BFIBSP8BIB | 1 | 0 |
| TATABOX3 | 1 | 0 |
| -10PEHVPSBD | 1 | 0 |
| EECCRCAH1 | 0 | 1 |
| INRNTPSADB | 0 | 1 |
| LTRECOREATCOR15 | 0 | 1 |
| WBOXHVISO1 | 0 | 1 |
| WBOXNTERF3 | 0 | 1 |
| WBOXNTCHN48 | 0 | 1 |
| WRKY71OS | 0 | 1 |
| CBFHV | 0 | 1 |
| IBOXCORE | 1 | 1 |
| ROOTMOTIFTAPOX1 | 1 | 1 |
| CACTFTPPCA1 | 3 | 1 |
| CAATBOX1 | 0 | 2 |
| GT1CONSENSUS | 0 | 2 |
| DOFCOREZM | 2 | 2 |
| GATABOX | 4 | 3 |

**Supplementary Fig. 1** ECL immunoblots of protein samples used for rBiFC analysis. Detected were only the nYFP proteins that also carried an HA-tag using a primary anti-HA-antibody. (A) Samples showing expression of BBX19_a_ fused with nYFP. (B) Samples of leaves showing expression of the BBX19 mutant (BBX19_h_) fused with nYFP. (C) Controls showing expression of unfused nYFP for the negative controls. Extracts from leaves that were not infiltrated gave no signal demonstrating the specificity of the applied antibody. The same amount of protein (40 µg) was loaded on each lane. Arrows indicate the specific protein fusion.


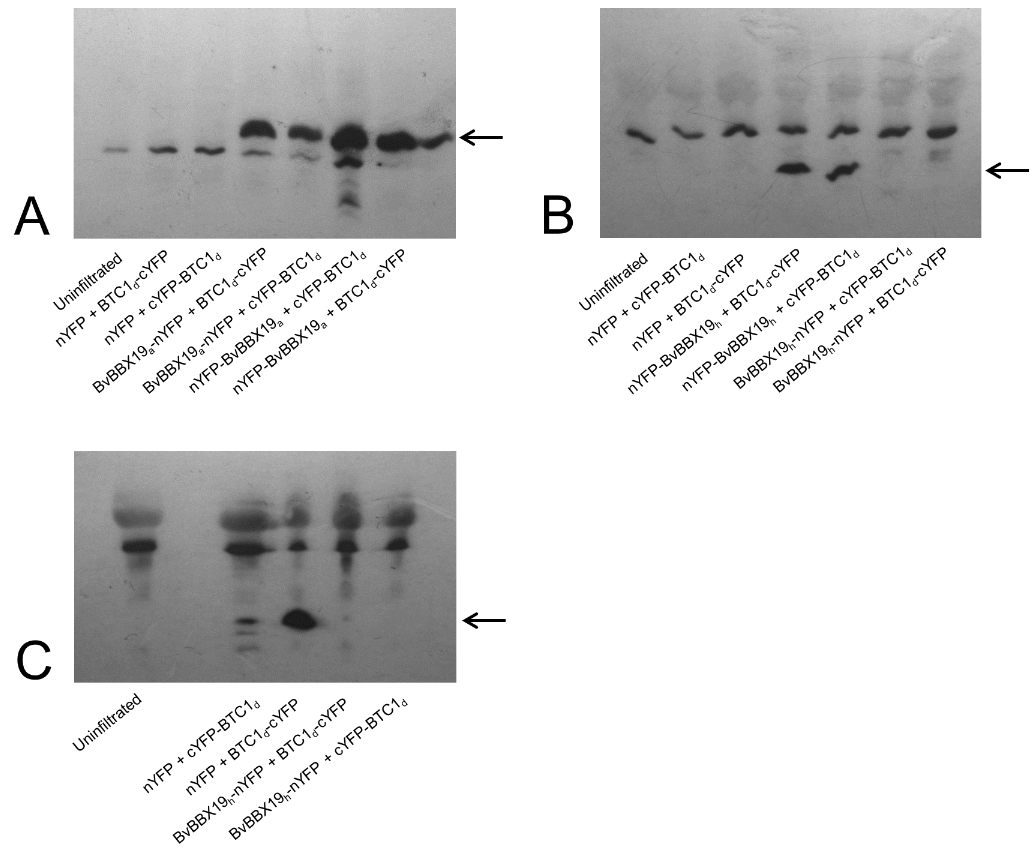


# References

1 Higo K., Ugawa Y., Iwamoto M. & Higo H. PLACE: A database of plant cis-acting

regulatory DNA elements. *Nucleic Acids Res.* **26**(1):358-359 (1998).
